# Supplementary material for: Are carbon emissions trading and green financial instruments synergistic? -Comprehensive quantitative research based on content analysis
Source: PLoS One. 2024 Mar 7;19(3):e0298601. doi: 10.1371/journal.pone.0298601 (PMC10919601; doi:10.1371/journal.pone.0298601)
Supplement: S1 Data — (ZIP) [file pone.0298601.s001.zip › Data/地级市Z_F工作报告.zip!/║╙─╧/─╧╤⌠╩╨/└┤╫╘╣1⁄2╓┌║┼í╛┬φ┐╦╩2╛▌═°í┐_2012.docx]

各位代表:

现在，我代表市人民政府，向大会作工作报告，请予审议，并请各位政协委员和其他列席人员提出意见。

折叠编辑本段主要内容

一、去年工作回顾

加快转型发展，综合实力迈上新台阶。初步测算，全市生产总值完成2229亿元，比上年增长11.2%。财政总收入155亿元，增长26%，其中，一般预算收入87.1亿元，增长26.1%。财政总支出307.8亿元，增长24.6%。工业强市实现新突破。全年工业增加值突破1000亿元大关，其中，规模以上工业完成增加值810亿元、增长21.8%，实现利润180亿元、增长50.4%。106个重点工业项目完成投资129亿元。六大战略支撑产业增加值对规模以上工业增长贡献率达到71%，三大战略新兴产业实现产值70亿元。产业集聚区建设步伐加快，14个产业集聚区完成投资858亿元，投资总额跃居全省前列。大力淘汰落后产能，万元生产总值能耗下降3.5%左右。农业基础地位进一步强化。粮食总产达118.7亿斤，连续8年增产。高效特色农业快速发展，畜牧业产值占农业总产值比重达到1/3。加大农业基础设施投入，创建农业标准化示范项目3个，改造中低产田28.5万亩，鸭河口水库除险加固工程如期完成，石步河水库建设顺利推进。植树造林94.2万亩。第三产业逐步壮大。全社会消费品零售总额930亿元，增长17.8%。镇平玉文化改革发展试验区、社旗赊店商埠文化产业示范区建设成效明显，全年新增4A级景区3个，接待游客2300万人次，实现旅游总收入120亿元。金融机构各项贷款余额973亿元，增长18.6%。南阳市商业银行成功更名为南阳银行，南阳村镇银行县市区网点布局实现全覆盖。

统筹城乡发展，城乡面貌发生新变化。中心城市发展取得新突破。全年城市建设新开工项目总投资超过50亿元，相当于过去10年投资的总和。新建改建城市主干道40余条、跨白河大桥3座，顺利完成75条背街小巷改造，开工24个城中村改造项目，完成邕河治理试验段整治和污水处理厂二期工程，供水、供气、供热和污水管网建设改造步伐加快。深入开展"两争一迎"活动，加强城市精细化管理，国家卫生城市创建取得阶段性成效，城市形象和品位大幅提升。南阳新区管理机构组建运行。鸭河、官庄工区规划建设有序展开。县城、小城镇、新农村建设步伐加快。县城建设力度加大，综合承载能力不断增强。一批特色镇建设品位明显提升。全年新农村建设投入资金8.6亿元，26个省级示范村规划建设全面铺开。城乡交通体系建设扎实推进。南阳机场二期改扩建工程进展顺利。开通南阳直飞杭州航线和直达上海始发列车。宁西铁路新增二线工程征迁前期工作启动。内邓高速建设和312国道、333省道改造顺利推进，三淅高速开工建设，建成干线公路235公里、农村公路808公里。

奋力攻坚克难，两大"国字号"工程谱写新篇章。南水北调中线工程移民迁安任务顺利完成。丹江口库区第二批8.6万移民实现平安、顺利、和谐搬迁，如期实现省委、省政府确定的16.5万移民"四年任务、两年完成"目标，创造了令人瞩目的"南阳速度"、"南阳经验"，被国务院南水北调办誉为世界水利移民史上的奇迹!移民后续稳定发展工作统筹推进。141个移民新村对新型农村社区建设起到了较好的示范带动作用。南水北调中线工程建设和水质保护稳步推进。总干渠征迁安置工作和谐高效，10.15万亩工程规划建设用地提前移交。工程建设进展顺利，渠道开挖成型已达40%以上。渠首地震安全项目高质量完成。实施库区及上游水污染防治和水土保持项目103个，库区森林覆盖率达到53%。农运会筹备全面提速。主体育场、游泳馆、综合训练馆等硬件建设大头落地，新闻中心、接待宾馆、通信、供电等配套基础设施建设进展顺利。吉祥物、会徽、主题口号揭晓，"即开型"体育彩票在全国发行。李宁体育公园开工建设。全民健身活动掀起热潮，我市被命名为国家"全民健身示范市试点城市"。深入开展迎农运主题教育活动，群众文明素质不断提升。赛事组织工作全面启动，全市迎农运氛围日益浓厚。

在服务保障两大"国字号"工程建设中，广大移民和征迁群众顾全大局，甘于牺牲，惜别故土;各级干部和建设者迎难而上，负重拼搏，任劳任怨，很多同志为此积劳成疾，有的甚至献出了宝贵的生命。他们付出的牺牲和奉献，祖国不会忘记，人民不会忘记，南阳不会忘记!他们的崇高精神，将永载史册，成为加快南阳发展的宝贵财富和巨大动力!

深化改革开放，发展动力活力进一步增强。改革不断深化。市属企业脱钩改制完成年度目标。医药卫生体制改革深入推进，在全省率先推行医院用药阳光集中配送新模式。公安警务机制改革取得阶段性成果。集体林权制度改革主体任务完成。盘活政府资产资源，创新融资模式，融资平台实力不断壮大。积极推进"421"企业上市计划，西泵公司、淅减公司成功上市。开放合作持续扩大。全年实际到位省外资金351亿元，实际利用外资3.89亿美元，分别增长93.7%、35.1%。成功举办第九届玉雕节和第十届张仲景医药科技文化节。与富士康的战略合作取得重大进展，数码投影机、LED等项目竣工投产。尤其是从烟台等地转移的五条LED生产线，从搬迁到达产仅用10天时间，速度之快令人震惊!富士康的到来，将强力拉动我市光电产业发展，更将产生巨大的"鲶鱼效应"，对南阳扩大开放、转型发展、企业经营、社会就业等方面带来广泛而深远的影响。与央企新签约的8个合作项目，总投资333亿元，居全省第二。中石化拟投资300亿元用于河南油田扩能改造和新项目建设，南阳产油、外地炼油的局面即将改变!

持续改善民生，和谐社会建设迈出新步伐。全市财政用于民生支出223.7亿元，增长33.4%。"十项重点民生工程"基本完成。城镇居民人均可支配收入、农民人均纯收入分别达到17289元和6776元。社会事业全面发展。优先发展教育事业。新建、改扩建中心城区中小学校12所，改造维修农村中小学校舍39.9万平方米。全市普通高招取得10年来最好成绩。职教攻坚稳步推进。文化惠民工程顺利实施。全国双拥模范城创建通过考评验收。第六次全国人口普查工作圆满结束。人口自然增长率低于全省平均水平。社会保障体系进一步健全。城乡居民社会养老保险制度试点县市区增加到9个，基本实现了养老保险全覆盖。医疗保险统筹层次和报销比例大幅提高，实现城乡全民医保。全年向上争取各类社会保障资金27亿元，惠及551.4万群众。新增各类就业25.3万人，城镇登记失业率控制在3.5%以内。开工建设2.47万套保障性住房，完成农村危房改造2.85万户。解决59.4万人饮水安全问题，新增农村沼气用户3.08万户，实现10.5万农村贫困人口脱贫。社会大局保持和谐稳定。安全生产形势平稳，食品药品监管有力，信访工作扎实有效。严打整治行动持续开展，网上追逃"清网行动"在全国保持领先位次，综合打防效能全省第一，人民群众安全感大幅提升。民主法治和精神文明建设进一步加强。坚持依法行政，自觉接受人大及其常委会的监督，支持政协参政议政，办理省、市人大代表建议和政协提案792件，办复率100%，满意率95.7%。坚持制度、教育、监督并重，反腐倡廉建设不断加强。广泛开展精神文明创建活动，公民道德水平和文明程度不断提升。同时，工商、质监、税务、司法、海关、检验检疫、审计、物价、民族宗教、史志、外事侨务、邮政、通讯、电力、残疾人、气象、人防、对台、国防教育等工作取得新成绩。

注重谋划运作，强化"四个带动"，跨越发展的势能和后劲不断积聚。谋划全局获得重大政策支持。《国务院关于支持河南省加快建设中原经济区的指导意见》中明确点到南阳9处、涉及南阳29处，诸多事关南阳未来发展的重大事项上升到国家层面。省政府出台《关于支持南阳市经济社会加快发展的若干意见》，开创了建国以来省政府出台文件支持单个省辖市发展的先河。24个省直部门相继出台了支持南阳发展的具体政策。项目、资金争取卓有成效。全市纳入省"十二五"规划重点项目2034个、总投资1万亿元，项目数和投资额分别居全省第一、第二。郑万铁路、蒙西至华中铁路列入国家"十二五"建设规划。南阳列入国家老工业基地调整改造范围。市县(部门)联动，向上争取资金支持，实现财政支出由200亿到300亿的跨越。"四个带动"带出新局面。实施项目带动，省市241个重点项目完成投资342.5亿元，全社会固定资产投资完成1477.7亿元、增长25.4%。实施品牌带动，坚持质量兴市，全年新创国字号品牌21个、省级品牌129个，评选首届南阳知名品牌100个。实施创新带动，启动实施中英低碳技术合作项目，成功创建车用生物燃料技术国家重点实验室，科技进步对全市经济增长的贡献率突破50%。实施服务带动，"转方式、正风气、提效能"活动取得明显成效，建成市行政效能电子监察系统，取消行政许可项目68项，减少行政事业收费项目170项，审批时限平均缩短6.7个工作日。

各位代表!2011年是"十二五"规划起步之年，也是我市奋力攻坚、克难前行的一年。这一年，我市经济社会发展在多重挑战下保持了好的趋势、态势和气势，实现了粮食生产、工业强市、城市建设、招大引强、争取政策支持等方面新的突破，完成了天下第一难、时限提前两年的移民迁安硬任务，打好了穷市办大事、建设任务相当艰巨的农运会筹备攻坚战!这一年，我市确立了建设开放富裕魅力和谐新南阳的总目标，明晰了建设中原经济区南阳主体区、突出"四个着力"、实现"四个突破"、加快"五区"建设的发展方向、路径和保障。这一年，我们在实践中深切体会到，推进政府工作，必须站位高远谋发展，集中突破带全局，勇于攻坚敢担当，强化运作求实效，执政为民惠民生。

成绩来之不易，积累弥足珍贵。这是省委、省政府和市委正确领导的结果，是市人大、市政协积极支持的结果，是全市广大干部群众共同奋斗的结果。在此，我代表市人民政府，向工作在全市各条战线上的工人、农民、知识分子、干部、人民解放军指战员、武警消防官兵、公安民警以及各界人士表示崇高的敬意和衷心的感谢!

在充分肯定成绩的同时，我们也清醒地看到:南阳发展相对落后的现实没有根本改变。南阳大而不强的基本市情没有根本改变;经济增长速度不快的局面没有根本改变;经济结构不优、城镇化水平和对外开放度偏低的状况没有根本改变。制约经济发展的瓶颈没有有效破解。市区管理体制不顺、中心城区带动能力弱、事业单位和国有流通企业改革滞后，制约发展的体制机制性矛盾突出。资金、土地、节能减排等要素约束和煤、电、油、运保障压力加大。影响社会建设的突出问题有待解决。社会事业欠账较多，一些关系群众切身利益的问题尚未得到妥善解决，安全生产、食品安全、社会治安等方面还存在不少问题，维护稳定的任务仍很艰巨。服务保障大局的挑战十分严峻。两大"国字号"工程建设的繁重任务及其形成的倒逼机制，对我们推动转型跨越发展、保障改善民生带来了巨大压力。政府自身建设还存在一些薄弱环节。政府效能不高，发展环境不优，一些干部的思想观念、素质和工作作风与南阳发展的要求和人民群众的期望相比还有很大差距。

各位代表!正视落后才能摆脱被动，破解难题方可赢得主动。我们将时刻保持清醒与忧患，振奋精神，迎难而上，积极寻求破解之道，以更大的魄力和胆识，以更足的锐气和干劲，奋力谱写南阳发展的新篇章!

二、今年政府工作的总体要求和目标任务

今年是实施"十二五"规划承上启下的重要一年，也是加快建设中原经济区南阳主体区、筹办第七届全国农运会和服务保障南水北调中线工程建设的关键之年。审时度势，我市面临的宏观经济形势更为复杂严峻，区域竞争日趋激烈，同时又担负着保护水质和生态、维护粮食安全等重大任务，在探索和推进"两不牺牲"、"三化"协调科学发展方面面临巨大压力。但国内外产业向中西部加快转移的趋势没有改变，国家积极的财政政策和稳健的货币政策继续实施，中原经济区建设全面推进对我市借势发展带来的重大机遇日益凸显，省政府9号文件全面落实的政策支持力度不断加大，两大"国字号"工程综合带动效应持续扩大。我们既要正视困难和挑战，更要看到机遇和优势，坚定必胜信心，开拓奋进，努力推动南阳经济社会跨越式发展。

今年政府工作的总体要求是:以科学发展为主题，以加快发展方式转变为主线，以扩大开放为主旋律，强力推进中原经济区南阳主体区建设，突出抓好两大"国字号"工程、"五区"发展和以重大项目建设为引领的"四个带动"，奋力走出一条不以牺牲农业和粮食、生态和环境为代价的"三化"协调科学发展路子，为建设开放富裕魅力和谐新南阳奠定坚实基础。

今年我市经济社会发展的主要预期目标是:生产总值增长11%，规模以上工业增加值增长16%，公共财政预算收入增长12%，全社会固定资产投资增长22%，社会消费品零售总额增长17%，进出口总额增长31%，实际利用外商直接投资增长21%，城镇居民人均可支配收入和农民人均纯收入均实际增长9%，居民消费价格指数控制在4%左右，节能减排完成省定目标。

实现上述要求和目标，做好今年工作，关键是把握好以下几点:一是必须充分融入中原经济区全面建设的洪流之中，紧扣"两不牺牲"、新型"三化"协调科学发展核心，加快构建"十大战略支撑体系"，在建设中原经济区南阳主体区上迈出重大步伐。二是必须把加快转型、跨越发展作为富民强市的第一追求，着力强产业、创优势、破瓶颈，努力形成"加快"、"跨越"、"率先"之势，尽快扭转南阳发展在全省及周边相对落后的被动局面。三是必须实施开放主战略，强化项目支撑，深化"四个带动"，以大开放促进大发展，以大上项目、打造品牌、推进创新、优化服务带动大发展。四是必须把改善民生作为政府一切工作的出发点和落脚点，千方百计办好涉及民生的实事好事，让人民群众真正成为发展改革成果的受益者。

重点抓好以下十个方面工作:

(一)强化项目带动，支撑经济社会转型跨越发展

项目决定结构，投资支撑发展。项目建设承载着南阳的未来和希望，必须以项目论英雄，努力形成重视项目就是重视发展、推动项目就是务实重干、落实项目就是执政有力的浓厚氛围和鲜明导向。牢牢抓住项目带动这个总抓手，大力开展"项目建设提升年"活动，不断开创大上项目、上大项目、上好项目的新局面，为经济社会转型跨越发展提供强大支撑。加快重点项目建设。全年力争投资超亿元项目425个，总投资2735亿元，年度计划投资820亿元。在产业发展、基础设施、城市建设、文化工程、生态建设等领域，重点抓好一批对南阳经济社会发展全局具有引领性、支撑性、带动性的重大项目，带动全市固定资产投资突破1800亿元。突出抓好与富士康、中石化两大战略合作项目，全力服务支持富士康更多项目落户南阳，并加速扩大产能，尽早形成辐射带动效应;全力服务支持河南油田扩能改造和改善企业生产生活条件。加快推进新能源汽车项目研发和建设、天冠集团纤维乙醇产业化、龙成集团煤清洁高效利用、防爆集团电机专业化生产中心、河南三源粮油加工、乐凯华光印刷产业基地、南阳煤炭储备基地、李宁体育公园和方城、唐河、社旗风电等重点项目建设进度。争取天池抽水蓄能电站尽早开工，加快推进鸭电三期、宛西电厂、白河南热电厂、邓州热电厂、镇平火电厂等前期工作。搞好项目谋划争取。认真研究国家政策导向和省投资重点，围绕产业、资源等优势，精心筛选和储备一批成型项目，建立前瞻性、针对性、操作性强的项目库。加强对接汇报，争取更多项目和资金。破解项目落地瓶颈。强化集约节约用地，盘活存量土地，加大建设用地报批力度，统筹安排好用地指标，保障重点项目用地需求。鼓励引导金融机构加大项目建设信贷支持力度，加强融资平台和中小企业信用担保中心建设，创新融资方式，多渠道解决项目建设资金问题，缓解企业融资困难。加快实施职教攻坚和全民技能振兴工程，为项目建设提供人才保障。

(二)实施开放带动主战略，深入推进改革创新

封闭保守，寸步难走。南阳发展相对落后的根本原因是对外开放不够，改革、创新不足。实现南阳崛起，必须强力组织对外开放大突破、推动改革创新大跨越。全面深化对外开放。把扩大开放作为决定南阳前途命运的关键抉择和带动全局的战略性举措，进一步解放思想，增强开放意识，强化开放措施，全方位、多层次、宽领域扩大对外开放，努力建设内陆开放高地。强力推进大招商活动，更加积极主动地承接产业转移，围绕培育壮大优势产业，突出招大引强，着力引进一批产业关联度高、辐射带动能力强的龙头企业和大个项目。开展"招商引资项目落地年"活动，强化签约项目跟踪落实，力争全年利用外资4.2亿美元、引进省外资金400亿元。加强企业战略合作，充分发挥与央企之间的历史合作渊源优势，进一步推动与中国兵总、中石化、首钢控股、国药集团、中国航天等央企的战略合作。打造开放平台，加快南阳出口加工区、南阳机场航空一类开放口岸争取步伐。完善工作机制，强化政策激励，提升服务水平，优化投资环境，实现真正意义上的举市开放。强力推进各项改革。深化国有企业改革改制。做好国企改革遗留工作，基本完成市属企业脱钩改制，加快建立现代企业制度，促进企业增强活力、提高效益，进入快速发展轨道。推进行政管理体制改革。全面实施事业单位岗位管理、公开招聘制度。稳妥推进警务机制改革和监狱体制改革。做好省直管县和经济发达镇管理体制改革试点工作。积极推进预决算公开。继续深化农村改革。全面开展农村集体建设用地流转工作。巩固集体林权制度改革成果，启动配套改革工作。深入推进水利工程管理体制改革。加快推动非公有制经济、县级供销社、资源价格、金融、环保、文化等领域的改革。加快创新型南阳建设。加强科技创新平台建设，支持南阳国家高新区建设国家创新型特色园区、南阳新能源产业集聚区建设省级创新型产业集聚区，争取中光学集团、宛西制药企业技术中心晋升国家级企业技术中心，提升产业科技创新能力。争创国家级创新型企业(试点)1家、新认定高新技术企业3家。争创国家创新型试点城市、国家知识产权示范市和国家科技进步示范市。

(三)强化新型城镇化引领，推进城乡一体化发展

加快推进新型城镇化，是破解南阳人多地少矛盾、解决"三农"问题、缩小城乡差距、推动转型跨越发展的必由之路。细化提升全市城镇规划体系，全面实施城乡建设三年提升行动计划，统筹推进中心城市、县城、小城镇、新型农村社区建设，加快构建具有南阳特色的现代城镇体系，引领"三化"协调发展。

加快中心城区发展步伐，增强龙头带动作用。围绕建设山川秀美、文化灿烂、经济繁荣、生态宜居的豫鄂陕省际区域性中心城市，高起点规划、高水平设计、高标准建设、高效能管理，着力做大做强做靓中心城区。搞好城市规划。规划是新型城镇化的生命所系、成败关键，必须高度重视、加大投入，以高水准的规划设计引领城市建设与发展。修订完善城市总体规划，加强分区规划、专项规划、近期建设规划和重点区域设计，力争年底前实现高标准的控制性详规全覆盖。完成中心城市组团发展规划编制，推进中心城区与官庄、鸭河工区和镇平、社旗、唐河等县城组成半小时城市圈，实行组团化发展;推动中心城市内部板块式组团发展，加快南阳新区、三个产业聚集区、东北分区、高新片区、老城片区、麒麟片区、武侯片区等9个功能组团规划建设步伐。加快编制中心城区水系建设规划，构建以白河景观轴为核心、以南水北调中线工程干渠和白桐干渠为两翼、以城区内河为脉络的大水系景观。严格规划执法检查，深入开展违法占地和违法建设综合整治，切实维护规划的严肃性和权威性。完善城市功能。全面推进城市交通、供排水、垃圾污水处理、供热、供电、供气、公共卫生、生态等基础设施建设，合理布局建设教育、医疗、文化、休闲娱乐等公共服务设施和环保、商业服务设施，全年完成投资30亿元以上。力争光武大桥"五一"建成，雪枫大桥6月底通车，312国道中心城区段改线7月底完工，独山水厂3月底竣工投运。继续推进城中村和旧住宅小区改造。深化提升中心城区背街小巷改造工作。规范城市路名。把公共交通建设作为完善城市功能、关注民生的重要任务，高度重视，提早谋划，科学设计，加大投入，强力推进，持续开展交通建设和整治，使南阳真正成为一个群众出行方便、交通有序的畅通城市。优先发展公交，农运会之前新增出租车300台、公交车100台。加快内河截污进程，逐步实施雨污分流，基本完成邕河治理任务，启动其他内河治理工程前期工作。完成白河台地景观区、迎宾大道、农运会场馆区域绿化等建设任务，提升独山森林公园形象，高标准建设城市外围生态防护林体系。实施区块突破。强力推进南阳新区发展，加快规划编制，创新管理体制，鼓励先行先试，强化招商引资，加快核心起步区建设步伐，确保实现一年打基础、三年出形象、五年成规模。加快官庄、鸭河工区发展，确保在基础设施建设、招商项目落地等方面取得突破性进展。进一步明确、理顺市区两级事权，鼓励支持卧龙、宛城、高新区加快发展。围绕农运会主体育场、新闻中心和体育中心三"核"，完善提升东北分区。研究出台中心城区经济振兴工程规划和实施意见，加速壮大城市经济。依托新能源、光电和高新技术产业集聚区，大力发展先进制造业和现代服务业。完善城区经济发展载体，规划建设一批提升城市功能品位的城市综合体、商务中心区和特色商务、IT产业等区(街)，形成一批服务业产业集群，培育城区经济新的增长点。积极发展郊区特色高效农业，打造一批特色高效农业示范园和农游一体化基地。

强化县域城镇承载承接作用和新型农村社区战略基点作用，大力发展县域经济。支持邓州建设南阳副中心城市，尽快把其他县城发展成为20万以上人口的中等城市;以"争星创强"为载体，加快小城镇建设步伐，大力发展沿线、沿边城镇带和50个强镇，引导支持基础好的中心镇发展成为小城市;把新型农村社区作为推动新型城镇化的战略基点，坚持多元投入，加强政策支持，实行典型引路，启动100个新型农村社区建设，各县市区选择1-2个乡镇，作为新型农村社区整体推进乡镇，先行先试，确保年内初见成效。研究出台加快壮大县域经济的政策措施，支持各县市加速发展。

构建城乡一体化发展新格局。促进城乡公共服务一体化。统筹推进城乡规划、产业发展、基础设施建设、公共服务、劳动就业和社会管理，推动城乡经济协调发展和基本公共服务均等化，切实解决进城农民就业、户籍、住房、社会保障、子女入学等问题，逐步使符合条件的农民转为城镇居民。加快综合交通运输体系建设。强化南阳区域综合交通枢纽地位，完成南阳机场二期改扩建工程;争取宁西铁路增建二线工程早日开工;加快推进郑万铁路、蒙西至华中铁路、南阳火车站改扩建工程前期工作，争取郑万铁路郑州至南阳段先行启动;加快推进内邓高速和三淅高速、312国道改造、231省道大修等工程建设，争取开工建设武陟至西峡高速公路南阳段。完成社旗、新野、淅川3条高速连接线和邓州至渠首连接线项目建设，实现所有县城20分钟上高速目标。改善火车站窗口形象，启动南阳中心汽车站改扩建工程，开工建设汽车北站和方城、桐柏等运输场站，推进干线客运、城乡公交、城市出租、农村客运一体化。

(四)强化新型工业化主导，加快转型升级，提升支撑能力

工业兴，则南阳兴;产业强，则南阳强。加快推进新型工业化，是加快生产方式转变、实现由传统农业大市向经济强市跨越的决定性力量。坚持做大总量、优化结构并重，制造业与服务业融合，构建结构合理、特色鲜明、节能环保、竞争力强的现代产业体系，支撑"三化"协调科学发展。实施工业强市战略。积极推进产业产品结构和企业组织结构调整，以大项目为支撑，集中培育大产业、大集群、大企业，强化科技支撑和服务保障，推动工业经济向更大规模、更高水平发展。加速做大做强优势产业。实行龙头带动、项目支撑、高端突破、延伸链条、集群发展，分项编制产业规划，出台配套政策，组建专门推进班子，加速做大做强装备制造、电力能源、冶金建材、油碱化工、纺织服装、食品加工六大战略支撑产业和新能源、新材料、光电三大战略新兴产业，重点抓好126个工业转型升级项目，力争装备制造、冶金建材、纺织服装3个产业实现营业收入均突破500亿。狠抓产业集聚区建设。把壮大规模和培育主导产业、拉长产业链条、形成产业集群结合起来，推广南召产业集聚区培育主导产业的做法，开展产业集聚区上档升级活动，力争在全省评比考核中升位争先。努力实现西峡产业集聚区主营业务收入超300亿元，高新区、新能源产业集聚区超200亿元，邓州、淅川、新野等产业集聚区超百亿元，力争全市产业集聚区固定资产投资完成1000亿元以上，实现主营业务收入1500亿元以上。着力培育优势骨干企业。继续抓好工业企业"双百"工程，选择一批重点骨干企业，实行重点扶持和动态管理，促其快速发展。大力支持龙成集团等百亿企业进一步做大做强。推动天冠、淅铝、新纺主营业务收入向超百亿目标迈进，加快西保、防爆、淅减、二机石油、中源化学、中光学、乐凯华光、中南钻石、中联水泥南阳分公司等骨干企业向超五十亿目标迈进。实施中小企业"小强人"培育计划，筛选50家高成长型中小企业进行扶持，壮大工业第二梯队。强力实施"421"企业上市计划，力争全年新增上市企业3-4家，尽快消除上市企业空白县市区。

壮大提升服务业。服务业是保障工业、带动农业、做强城市的重要支撑。坚持生产性服务业与生活性服务业并重，完善、细化服务业专项规划，研究出台激励政策，促进服务业加快壮大、提升。大力发展现代物流、金融保险等生产性服务业。加快区域性物流枢纽规划建设，引导物流业规范有序发展。引进、培育大型物流企业，重点发展工业品、农产品、建材、汽车、摩托车配件及冷链物流，突出抓好美的工业物流园、中商农产品中心批发市场、汇融物流园区、南阳农机五金机电物流园等项目建设。积极引进各类金融保险机构在宛设立分支机构，加快驻宛股份制银行增设网点设立步伐，争取南阳银行跨区域布局、县级农信社改革组建农村商业银行实现突破，积极发展保险、证券、担保等金融业态，优化金融生态环境，防范化解金融风险，努力打造区域金融高地。积极培育科技研发、创意设计、信息服务、服务外包、会展等新兴服务业态。发展壮大旅游、传统商贸、房地产、餐饮、住宿等生活性服务业。提升壮大旅游产业。抓好中心城区历史文化名胜、恐龙遗迹园、宝天曼、桐柏淮源、莲花温泉水城等景区深度开发和标准化管理。大力发展乡村休闲旅游。改造提升伏牛山生态旅游环线。加快建设区域性游客服务中心。推进旅游区域合作，加快南水北调中线工程生态文化旅游带建设。加大旅游宣传推介力度，加快旅游产品开发，提高从业人员素质。力争全年接待游客2900万人次，实现旅游综合收入150亿元。规范提升商贸服务业。编制实施中心城区商业网点总体布局规划和专项规划。加快中心城区农贸市场、便民菜店的规划建设，推动标准化菜市场改造升级。大力推进"万村千乡市场"工程。促进房地产业健康发展，大力引进土地市场一级开发企业和全国排名前50位的房地产企业，增加住房有效供应，抑制房价过快上涨。积极发展健康医疗、社区服务、养老服务、家庭服务等新型业态。

(五)强化新型农业现代化基础，持续做好"三农"工作

现代农业是新型城镇化和新型工业化的重要基础，更是农民就业、致富的有力支撑。必须珍视和发挥南阳农业的巨大优势和潜力，始终把"三农"工作摆在重中之重的位置，紧紧围绕"保粮食、调结构、促增收"目标，实行示范带动、项目支撑、科技引领、重点突破，抓好粮食生产，培育壮大畜牧、蔬菜、花卉、茶叶、种苗、中药材等高效特色产业，大力发展农副产品加工业，加快新型农业现代化步伐，努力使现代农业为南阳创造更多财富，为农民带来更多实惠，为农村带来更大繁荣。抓好示范区建设。加快推进卧龙农业国家高新科技示范园区、社旗农区新型"三化"协调发展试验示范区、邓州现代农业示范区、方城赵河农业综合改革试验区、新野肉牛产业集群建设。鼓励支持各县市区因地制宜，选择培育一个万亩粮食高产示范区、一个万亩高效特色农业示范区、一个畜牧产业化示范区，从而带动农业生产要素集约化、农业产业化、农民专业化、农区工业化、农村社区化。加快农业产业化进程。围绕我市农业区域布局，针对产业化经营中的薄弱环节，特别是农产品精深加工不足等问题，筛选确定100个政策性农业项目、100个农业招商引资项目和100家农业产业化龙头企业，大力发展农民专业合作社，带动农业产业结构调整优化，拉长农产品加工链条，提高农业整体效益。推进农业科技进步。确定全市农业十大科技推广项目、十大科技攻关项目、十大科技引进示范项目，助推农业上档升级，增强农产品市场竞争力。改善农村生产生活条件。继续开展粮食高产创建活动，大力实施高标准粮田"百千万"建设工程，规划建设一批百亩方、千亩方和万亩方高标准永久粮田，支持唐河县整建制推进粮食高产示范创建，力争全市粮食总产量达到120亿斤。加大农业综合开发力度，完成15万亩中低产田改造任务。加快农村水路电气等基础设施建设。加快中小型水库除险加固、中小河流治理及石步河水库建设步伐，实施灌区续建配套节水改造和小农水重点县项目，新增有效灌溉面积10万亩、除涝面积10万亩。加大扶贫开发力度，实施100个贫困村整村推进，解决10.78万农村贫困人口脱贫问题。

(六)全力筹备第七届全国农运会，办一届"高水平、有特色、重节俭"的农运盛会

9月份农运会举办在即，时间紧、任务重、要求高，全市人民充满期待，国家和省委、省政府寄予厚望，往届举办标杆很高，对我们形成了前所未有的挑战和考验。必须以强烈的紧迫感、使命感，举全市之力，坚决打赢这场万众瞩目的攻坚战。坚持节俭原则，全速推进软硬件建设。突出抓好新建场馆的设备安装和智能化建设，确保3月底进行测试赛。加快推进宾馆酒店软硬件建设，确保5月底全部投入试运营。加速推进竞赛信息化系统建设，同步跟进配套基础设施建设，打造"功能完备、保障有力"的竞赛环境。坚持精细化管理，确保赛事举办圆满成功。科学编制和实施竞赛、安保、交通、接待志愿者服务等各项赛事行动计划，严格时间节点，加强沟通协调，做到筹办工作无缝对接，实现安全、有序、文明办赛。坚持突出特色，狠抓开闭幕式策划组织和宣传造势工作。本着欢乐、祥和、节俭的原则，结合南阳人文优势，抓好开闭幕式策划组织工作。加大农运会宣传造势力度，精心策划好倒计时100天、30天等主题活动，营造全国迎农运的浓厚氛围，更好地扩大南阳影响、提升南阳形象，吸引社会关注南阳、支持南阳、投资南阳，拉动南阳经济社会发展。坚持"办赛事、惠民生"，放大农运综合效应。坚持市、县、乡、村四级联动，深化"两争一迎"活动，全面提升城乡环境面貌和群众文明素质。以建设全民健身示范市试点城市为契机，进一步健全完善城乡体育功能设施，大力发展休闲体育产业，推动全民健身活动常态化。加大农运资源开发和对口协作力度，实现借力发展。乘农运会举办东风，提高文化旅游业发展水平。超前研究后农运时期场馆、设施持续利用，充分发挥工程效益。

(七)扎实做好南水北调中线工程建设和移民后续工作，在服务保障大局中加快南阳发展

南水北调中线工程服务保障，是南阳当前和今后相当一个时期内的重大政治责任和艰巨历史任务。实现移民迁安，只是迈出了第一步，今后的任务会更加繁重。中线工程对南阳上下的大局观念、政治担当、领导方式、发展方式、为民意识等方面的要求和考验将长期存在。要深度挖掘压力和挑战中蕴藏的潜力和机遇，充分利用、发挥其形成的倒逼机制和综合带动效应，在服务大局中加快南阳自身发展。扎实做好移民后续稳定发展工作。按照"建班子、抓管理、保稳定、促发展"的思路，全力抓好移民安稳致富工作，改善移民生产生活条件。研究出台水源地生态移民规划和配套政策。全力服务保障好南水北调中线工程建设。完成干线征迁安置扫尾工作。深化"双创双评"活动，争创全线最优施工环境。尽早启动配套工程建设，确保与中线主体工程同步达效。切实做好水质保护。加快《丹江口库区及上游水污染防治和水土保持规划》项目实施进度，全面实行污染物总量控制，着力构建水源区生态建设屏障，完成造林114.5万亩。积极争取国家环保专项资金支持，加快推进渠首环保中心建设，开展农村环境连片整治，确保入库水质安全。抓好机遇，积极争取项目、资金支持，巩固扩大与京津等受水地区对口协作成果。

加快创建高效生态经济示范区。这既是南阳作为南水北调中线工程核心水源地义不容辞的责任，更是南阳立足生态优势，探索"两不牺牲"、"三化"协调科学发展之路的现实需要。加快高效生态经济示范区建设规划和实施方案编制，争取6月底前完成，尽快启动实施。创新发展模式，大力发展高效生态农业、生态工业和以生态旅游为主的现代服务业，致力打造生态产业体系;构建生态战略功能区，强力推进生态建设和节能减排，积极推动循环经济发展;加大环境保护力度，建设治污防护带，提高污染防治水平，全面创建南阳国家生态文明示范市，实现经济社会发展与生态环境保护有机统一。

(八)促进文化大发展大繁荣，不断提升发展软实力

南阳的悠久历史和灿烂文化，既是先人为我们留下的宝贵遗产，也是南阳人民自尊自信、自强不息的不竭源泉。文化的大发展大繁荣，关乎南阳的形象和环境，体现南阳的软实力和核心竞争力。认真贯彻落实党的十七届六中全会精神，充分挖掘南阳厚重的文化资源，努力实现南阳文化全面振兴，为中原经济区南阳主体区建设提供强大的精神动力和智力支持。加强思想道德建设。深入推进社会公德、职业道德、家庭美德、个人品德建设。总结发扬新时期南阳移民精神和农运精神，充分挖掘、宣传南阳本土涌现出来的英雄人物和先进典型。大力开展文明单位、文明市民、道德模范等评选表彰活动，深化拓展道德实践活动和群众性精神文明创建活动，在全社会形成积极向上的精神追求和健康文明的生活方式。完善公共文化服务体系。实施文化惠民工程。加快市县图书馆、文化馆和博物馆建设，推动文化信息资源共享、广播电视村村通等基层文化建设，推进公益性文化设施免费开放。深入开展文化、卫生、科技"三下乡"、"欢乐南阳"、"舞台艺术送农民"等活动，不断满足人民群众日益增长的精神文化需求。实施文化精品工程。挖掘、培养文化人才，推出更多富有南阳特色、具有全国影响力的精品力作。搞好文物、历史街区的保护和合理利用，加大非物质文化遗产保护力度，提升地方曲艺等本土传统特色文化的吸引力和感召力。加大知识产权保护力度，深入开展"扫黄打非"工作，净化文化市场。大力发展文化产业。修订出台南阳市文化发展规划纲要，做大做强文化旅游、玉文化、中医药文化等传统文化产业，加快发展文化创意、影视传媒等新兴文化产业。扎实推进卧龙岗文化旅游产业集聚区、镇平玉文化改革发展试验区、社旗赊店商埠文化产业示范区、西峡恐龙遗迹园区等重点项目建设，抓好以南阳府衙为中心的历史文化街区和以医圣祠为中心的张仲景·中医祖庭核心文化园区建设，培育一批具有重大示范效应和产业拉动作用的特色文化产业基地和龙头企业。扩大对外宣传和交流活动，推动南阳文化"走出去"，提升南阳文化的知名度和影响力。积极推动县市区有线电视网络整合，全面完成有线电视数字化整体平移。

(九)致力保障和改善民生，积极推进和谐南阳建设

我们的一切发展都是为了人民，一切奋斗都是为了让群众过上更加幸福的生活。始终秉持"民苦我忧、民贫我愧"之心，恪守为民之责，多办利民之事，千方百计改善民生，努力实现好、维护好、发展好广大人民群众的根本利益。加大投入，完善机制，继续办好"十项重点民生工程"，努力增加城乡居民收入，不断推进和谐社会建设，切实让人民共享发展成果。加快教育改革发展。加大教育投入，优化资源布局，大力发展各级各类教育。实施学前教育三年行动计划，逐步解决"入园难"问题。推进义务教育均衡发展，出台中心城区学校建设布局规划，加大中心城区中小学建设力度，缓解"上学难"、"大班额"问题。加快推进市一中分校规划选址和建设工作。结合事业单位改革，逐步把中心城区闲置教育资源整合转化为中小学、幼儿园。加强教师队伍建设，做好教师补进工作，推进城乡教师交流，实施"名师、名校长、名班主任"工程，全面提高教学质量。大力发展职业教育，加快职教园区建设步伐，搞好路网等配套基础设施建设。提升高校建设水平，推动南阳农业职业技术学院筹建、南阳医专升本和南阳幼师升专工作。大力发展医疗卫生事业。加快中心城区卫生设施建设，推进重大医疗卫生项目建设和优质医疗资源整合，高标准规划建设城市新区医院。完善城乡医疗卫生服务体系，推进社区医院建设，缓解群众"看病难"等问题。实施5个县级120急救指挥中心、10个县级卫生监督所和南阳市全科医生临床培养基地建设。积极支持中医药事业发展，整合优化中心城区中医医疗资源，实施县级中医院倍增计划。千方百计扩大就业。实施更加积极的就业政策，新增城镇就业9万人，失业人员再就业2.5万人，新增农村劳动力转移就业10万人。出台大力支持小型微型企业发展政策，鼓励全民创业，以创业带动就业。强化就业公共服务，重点抓好高校毕业生、城镇就业困难群体、退役人员和残疾人就业帮扶工作。构建和谐劳动关系，及时妥善处理劳动争议，完善职工权益保障机制，让劳动者更加体面地工作和生活。稳定增加城乡居民收入。落实国家、省调整收入分配关系的各项政策，努力增加城乡居民特别是低收入群众收入。完善机关事业单位工资和津补贴制度。进一步完善社会保障体系。实现城乡居民社会养老保险制度全覆盖，规范完善工伤、城镇基本医疗、生育、失业保险市级统筹。继续做好机关事业单位养老保险试点工作。巩固完善新农合制度。完善城乡社会救助体系。强力推进保障性安居工程建设，探索城中村开发项目配套建设机制，新开工建设各类保障性住房2.6万套，确保分配公开公正公平。妥善解决农运会场馆涉迁群众的安置问题。全面发展其他各项事业。加强公共体育设施建设，扎实做好"全民健身示范城市"试点工作。全面提高人口素质，优化人口结构，全年人口自然增长率控制在6‰以内。坚持军民融合发展，巩固发展双拥创建成果。切实做好统计、物价、税务、工商、外事侨务、对台、民族宗教、档案、地方志、气象等工作。建设和谐平安南阳。今年适逢党的十八大召开和第七届全国农运会举办，保持社会和谐稳定尤为重要。创新社会管理，探索新型社会组织、新型经济组织管理的新途径，提升社区建设和服务水平。高度重视信访工作，健全维护群众权益机制，积极预防、妥善处置突发事件和群体性事件。落实社会稳定风险评估制度，完善"大调解"工作机制。深入开展"安全南阳"创建活动，突出抓好重点行业、重点领域和重要设备的安全监管，坚决遏制重特大事故发生。加强食品药品安全监管，保障群众饮食用药安全。启动全市应急训练中心、人防疏散基地和16个应急避难场所建设。加强社会治安综合治理，严厉打击各种违法犯罪行为，建设平安南阳，增强群众安全感，促进社会和谐稳定。

(十)全面加强自身建设，努力建设人民满意政府

加快南阳发展，政府重任在肩。我们唯有牢记使命，直面挑战，勇于担当，奋力拼搏，创造佳绩，才能无愧于人民的重托和殷切期待。深入推进"两转两提"，认真落实"三具两基一抓手"，巩固提升"转方式、正风气、提效能"成果，努力建设法治、高效、为民、廉洁的人民满意政府。

坚持依法行政。严格依照法定权限和程序行使权力、履行职责。坚决执行人大及其常委会的决议决定，依法接受其法律监督和工作监督，主动接受政协的民主监督，支持政协参政议政，认真办理人大代表建议和政协提案，并积极探索有效机制。加强普法教育，推进综合执法，下移执法重心，减少执法层级，严格行政执法责任和过错追究。加强行政复议工作。深入推进政务公开，推行政府部门权力公开，让行政权力始终在阳光下运行。

提高政府效能。完善城市规划、土地储备、城市管理委员会等例会制度，定期研究解决重大和突出问题。加快推进公共资源交易中心建设，积极推行行政审批"两集中、两到位"，规范提升市县行政服务中心。完善重大事项、重点工作跟踪落实机制和激励评价机制，强力推进工作落实。推行政府绩效管理，强化行政问责，大力整治行政慢作为、不作为和乱作为，查处曝光一批典型案例，杜绝推诿扯皮、办事拖拉，切实提高办事效率。深入开展"创先争优"活动，设立政府工作贡献奖和创新奖，推动县市区在全省晋位升级、政府部门争当全省全国行业标兵，不断推出在全省乃至全国具有示范意义和富有效益的创新之举，形成干事创业的浓厚氛围和求实求效的价值导向。

恪守执政为民。牢固树立宗旨意识，把群众利益作为谋划工作的第一追求，把群众满意作为衡量工作的第一标准，真诚倾听群众呼声，真情关心群众疾苦，尽心尽力为群众做好事、办实事。认真解决企业改制、征地拆迁、环境保护、涉法涉诉等领域损害群众利益的突出问题，维护社会公平正义。积极开展争做"人民满意的公务员"活动，努力建设一支政治坚定、业务精湛、作风过硬、人民满意的公务员队伍。

严格廉洁从政。严格落实党风廉政建设责任制，扎实推进惩治和预防腐败体系建设，强化对权力运行的制约和监督。加大纠风和专项治理力度，强化对行政审批、招投标、土地出让、政府采购、产权交易等重点领域和关键环节的监管。厉行节约，反对奢侈浪费，严格控制行政经费等一般性支出。加强领导干部经济责任和政府投资建设项目审计。加大查处违法违纪案件力度，保持惩治腐败的高压态势，以廉政建设的实际成果取信于民。

各位代表!南阳发展正处在一个关键时期，跨越发展、后发争先，是时代和人民赋予我们的神圣使命。南阳没有时间可以等待，我们没有理由可以懈怠。让我们在省委、省政府和市委的坚强领导下，以更加振奋的精神、更加自觉的担当、更加务实的作风，锐意进取，奋发有为，圆满完成今年各项目标任务，奋力谱写南阳转型跨越发展的新篇章，以优异成绩迎接党的十八大胜利召开!
